# Expanding the genotype-phenotype spectrum in *SCN8A*-related disorders

**DOI:** 10.1186/s12883-023-03478-y

**Published:** 2024-01-17

**Authors:** Malavika Hebbar, Nawaf Al-Taweel, Inderpal Gill, Cyrus Boelman, Richard A. Dean, Samuel J. Goodchild, Janette Mezeyova, Noah Gregory Shuart, J. P. Johnson, James Lee, Aspasia Michoulas, Linda L. Huh, Linlea Armstrong, Mary B. Connolly, Michelle K. Demos

**Affiliations:** 1 Division of Neurology, Department of Pediatrics, BC Children’s Hospital, Faculty of Medicine, University of British Columbia, Vancouver, BC Canada; 2 Xenon Pharmaceuticals, 200–3650 Gilmore Way, Burnaby, BC V5G 4W8 Canada; 3 Department of Medical Genetics, BC Children’s Hospital, Faculty of Medicine, University of British Columbia, Vancouver, BC Canada

**Keywords:** *SCN8A*, Developmental and Epileptic Encephalopathy, Epilepsy, Seizure, Electrophysiological study, Variant of Uncertain significance, Exome sequencing, Loss-of-function, Gain-of-function

## Abstract

**Background:**

*SCN8A*-related disorders are a group of variable conditions caused by pathogenic variations in *SCN8A.* Online Mendelian Inheritance in Man (OMIM) terms them as developmental and epileptic encephalopathy 13, benign familial infantile seizures 5 or cognitive impairment with or without cerebellar ataxia.

**Methods:**

In this study, we describe clinical and genetic results on eight individuals from six families with *SCN8A* pathogenic variants identified via exome sequencing.

**Results:**

Clinical findings ranged from normal development with well-controlled epilepsy to significant developmental delay with treatment-resistant epilepsy. Three novel and three reported variants were observed in *SCN8A*. Electrophysiological analysis in transfected cells revealed a loss-of-function variant in Patient 4.

**Conclusions:**

This work expands the clinical and genotypic spectrum of *SCN8A*-related disorders and provides electrophysiological results on a novel loss-of-function *SCN8A* variant.

**Supplementary Information:**

The online version contains supplementary material available at 10.1186/s12883-023-03478-y.

## Background

Pathogenic genomic variations in *SCN8A* can cause a spectrum of neurological phenotypes characterized by developmental delay, early onset multivariate seizure types, intractable epilepsy, movement disorders and other neurological manifestations [1–3]. Psychomotor development varies from normal to abnormal from birth. Normal development may precede subsequent delay or regression following seizure onset. Variable degrees of intellectual disability is seen with ~ 50% having a severe form. Behavioral abnormalities are also seen in some individuals.

The expression of voltage-gated sodium channels (NaVs) is key for initiation and conduction of action potentials in excitable cells such as skeletal muscle and neurons [4]. Neurons typically express multiple NaV isoforms. Loss-of-function (LoF) and gain-of-function (GoF) of voltage-gated sodium channels can lead to a wide spectrum of phenotypes. *SCN8A* (NaV1.6; OMIM 600702) is one of nine human genes encoding voltage-gated sodium channel α-subunits more recently implicated in epilepsy [5]. *SCN8A* variants in patients with epilepsy primarily result in GoF in Nav1.6 and hyperexcitability of neurons in the central nervous system [6]. Evaluation of the phenotype and genotype spectrum in *SCN8A*-related disorders suggests that GoF mutations are associated with severe epileptic encephalopathy, while LoF mutations cause intellectual disability with or without seizures. Sodium channel-blocking agents are effective on different levels in the treatment of seizures in GoF mutations. Anti-sense oligonucleotide therapy is in clinical trials for GoF variants and several treatment modalities are being explored in research including transfected cell lines and mouse models [7]. Targeted and genome-wide next-generation sequencing (NGS) has significantly increased the number of families identified with *SCN8A-*related disorders, allowing scientists to prioritize functional studies and develop a better understanding of the phenotypic spectrum [3].

In this case series, we would like to add to the growing clinical and genetic data of over 500 individuals with *SCN8A*-related disorders by reporting 8 affected individuals with variable phenotypes including one family with a previously published variant associated with treatable epilepsy, as well as, novel variants in *SCN8A* identified by exome sequencing. We establish functional evidence for a LoF *SCN8A* variant by using electrophysiological analyses in a patient with intellectual disability, autism spectrum disorder, and abnormal EEG. The patient also presented a co-occurring variant of unknown significance in *KCNQ3.*

## Methods

Six families seen at neurology clinic, British Columbia Children’s Hospital were enrolled in the study. Exome sequencing was performed on the probands. Informed consent was obtained for the use of clinical and research findings for publication. The study has the approval from Institutional Ethics Committee (protocol number H14-01531). Clinical and molecular details of patients are summarized in Table 1. Detailed case description can be found in the Additional file 1.

**Table 1:** Clinical and molecular features of subjects with *SCN8A* variations

	Patient 1	Patient 2	Patient 2 - sibling	Patient 2 - father	Patient 3	Patient 4	Patient 5	Patient 6
**Age (first seen)**	4 months	3 months	2 weeks	NA	14 months	3 years	6–7 months	10 years
**Age (last seen)**	17 yrs	7 yrs	4 yrs	40 yrs	6 yrs	7 yrs	8 yrs	20 yrs
**Ethnicity**	Caucasian	Caucasian	Caucasian	Caucasian	Caucasian	Caucasian	Indian	Indian
**Sex**	Female	Female	Male	Male	Female	Female	Male	Female
**Family history**	Paternal cousin with epilepsy	Father and brother with same condition	Sibling of Patient 2	Father of Patient 2	Father with 2 febrile seizures, on valproic acid till 4 years of age	NA	None	None
**Epilepsy diagnosis**	DEE	Treatment responsive	Treatment responsive	Treatment responsive	Treatment responsive	Unclassified	DEE	Treatment responsive
**Genetic diagnosis (** ***SCN8A*** )*	NM_001330260.2:c.1238C > A (p.(Ala413Asp)	NM_001330260.2:c.5630A > G (p.Asn1877Ser)	NM_001330260.2:c.5630A > G (p.Asn1877Ser)	NM_001330260.2:c.5630A > G (p.Asn1877Ser)	NM_001330260.2:c.4447G > A (p.Glu1483Lys)	NM_001330260.2:c.971G > A (p.Cys324Tyr)	NM_001330260.2:c.773C > T (p.(Thr258Ile))	NM_001330260.2:c.986A > G (p.(Asp329Gly))
**Zygosity**	Heterozygous	Heterozygous	Heterozygous	Heterozygous	Heterozygous	Heterozygous	Heterozygous	Heterozygous
**CADD score**	29	25.4	25.4	25.4	31	27.2	26.5	25
**Inheritance**	De novo	Paternal	Paternal	NA	De novo	De novo	De novo	De novo
**Function**	Unknown	Gain-of-function	Gain-of-function	Gain-of-function	Gain-of-function	Loss-of-function	Unknown	Unknown
**Birth history**	Uneventful	Maternal preeclampsia without complications	Maternal preeclampsia without complications	NA	Uneventul	NA	Maternal gestational hypothyroidism, diabetes and hypertension	Uneventful
**Novelty of the variant (ClinVar Variation ID)**	Novel unreported	Known (130252), Anand 2016, Parrini 2016, Butler 2017	Known (130252), Anand 2016, Parrini 2016, Butler 2017	Known (130252), Anand 2016, Parrini 2016, Butler 2017	Known (253195), Gardella 2016	Known (1699202), Encinas 2020	Known unreported (559632 - submitter BCCHR)	Known unreported (1518887)
**Intellectual disability**	Profound	None	None	None	Normal	Yes	Yes	Normal
**Development**	GDD	Normal	Normal	Normal	Normal	Severe GDD	GDD	Normal
**Age at seizure onset**	4 months	3 months	7 months	6 months	12 months	uncertain	6–7 months	10 yrs
**Seizure type at onset**	IS, starring spells, hypsarrhythmia	Sudden arching of back, stiffness, choking sounds, frothing, greyish discoloration of the body	Stiffness, clicking sounds with eyes closed, body turned light gray	NA	Tonic stiffening with superimposed tremor with eyes open and unresponsive	possible clinical seizure	Arrest of activity with eyelid fluttering lasting for a few seconds	Head and eye deviation to the left, face pulling to the left, arched back and falling to the left side slowly to the ground, impaired awareness
**Course of seizure**	LGS manifesting impaired awareness seizures, atypical absence seizures, GTCS, epileptic spasms, non-convulsive status epilepticus	Self resolving focal seizures followed by fatigue	Focal seizure	NA	Focal seizures. A couple of episodes occurred along with febrile illness.	-	Absence seizures	Focal seizures with impaired awareness
**Age at EEG**	12 yrs	15 months	NA	NA	18 months	1 year - Normal	1.5 yrs	14 yrs
**EEG finding**	Slow dysrhythmic background, bifrontal slowing, frontal spike and slow wave, L > R, left occipital sharp and slow waves, no subclinical seizures	Normal	NA	NA	Normal	2 yrs - Dysrhythmic basal ganglia occasional generalized paroxysmal delta waves in sleep, frequent sharp waves in L temporal in drowsy/sleep with dipole configuration	Frequent generalized fragmentary spike and wave and atypical spike and wave activity maximal in the bilateral anterior quadrants of drowsiness and sleep	Normal
**Duration of seizure**	45min	2-6min	3min	NA	2-3min	-	3sec	10-15sec
**Frequency of seizure**	2–3 a day	2–3 a week	1 episode	NA	3–4 a month	-	1–2 a day	3 a month
**Seizure control**	Treatment resistance for many years. Now under control.	Seizure-free	Seizure-free	Seizure-free	Seizure-free	No clinical seizures	Good	Seizure-free
**Other clinical findings**	Hyperkinetic movements (choreo-athetoic), hypotonia, cortical visual impairment, sleep disorder, non-verbal, scoliosis, spastic quadraparesis, strabismus, G-tube fed	None	None	None	None	Overriding toes, drooling, microcephaly, corrected strabismus, far sighted, zoning out spells, one episode of lethargic, febrile, shaking, ?eyes rolling back	Previous history of ataxic gait	Hyperkinetic movement disorder, previously evaluated for daytime enuresis as a child
**Behavioral abnormalities**	None	None	None	None	None	Autism. Behavior described as impulsive with high degree of motor activity.	ADHD	None
**Treatment response**	Good	Good	Good	Good	Good	-	Good	Good
**Current treatment**	Clonazepam	Carbamazepine	Carbamazepine	Carbamazepine	None. Weaned off topiramate.	None	Ethosuximide, acetazolamide, biphentin	Carbamazepine
**Past ASM**	Vigabatrin, ACTH, carbamazepine, lamotrigine, leviteracetam, nitrazepam, oxcarbamazipine, topiramate, valproic acid, clonazepam, phenytoin, CBD oil, ketogenic diet	None	None	He was tried to wean on carbamazepine twice at 5 yrs and 19 yrs but failed both times. Now he takes 400 mg twice daily	Topiramate, clobazam	Valproic acid improved EEG abnormality	Lamotrigine, levetiracetam	None
**Brain MRI**	White matter volume loss and delayed myelination	Normal	-	NA	Normal	-	Mild nonspecific T2 hyperintense changes within the peritrigonal white matter	Normal
**Age at brain MRI**	2 yrs	8 months	-	NA	2 yrs	-	4 yrs	10 yrs
**Chromosomal microarray**	Normal	Normal	-	-	Normal	Normal	-	-
**Other relevant investigations**	Newborn screening, plasma and CSF amino acids and lactate were normal	Incidental homozygous benign BTD variant. Acylcarnitine screen, metabolic screen with plasma amino acids, newborn screening were normal	CBC normal	NA	-	Additional heterozygous, *de novo*, missense VUS in *KCNQ3*, NM_004519, c.1120C > G (p.Pro374Ala) (CADD 25.7)	-	Ultrasound renal - small echogenic abnormality just to the right of the midline within the bladder dome. This may represent an urachal remnant.

Abbreviations: *Genome Assembly GRCh37, DEE Developmental and epileptic encephalopathy, GDD Global developmental delay, IS Infantile spasms, LGS Lennox Gastaut syndrome, L Left, R Right, ASM Anti-seizure medications, ACTH Adrenocorticotropic hormone, CBD Cannabidiol, GTCS Generalized tonic-clonic seizures, BTD Biotinidase deficiency, NA Not available, CBC Complete blood count, - Not done, EEG Electroencephalogram, VUS Variant of unknown significance, VPA Valproic acid, ADHD Attention-deficit hyperactive behavior

### Exome sequencing

Exome sequencing was performed in all the families. Detailed methodology and steps followed for exome sequencing wet lab and data analysis has been previously described [8]. Sanger sequencing to validate the variants and to determine the segregation in the families was performed [9].

### Functional validation of *SCN8A*

The functional consequence of the *SCN8A*, c.971G>A (p.Cys324Tyr) variant was examined in vitro by heterologous protein expression in Human Embryonic Kidney cells (HEK-293). The electrophysiological properties of the HEK-293 cells expressing the p.Cys324Tyr protein were compared to control cells expressing either the wild-type protein or empty expression vector. Functional studies were not performed for the *KCNQ3* variant in Patient 4.

## Results

**Fig. 1:**
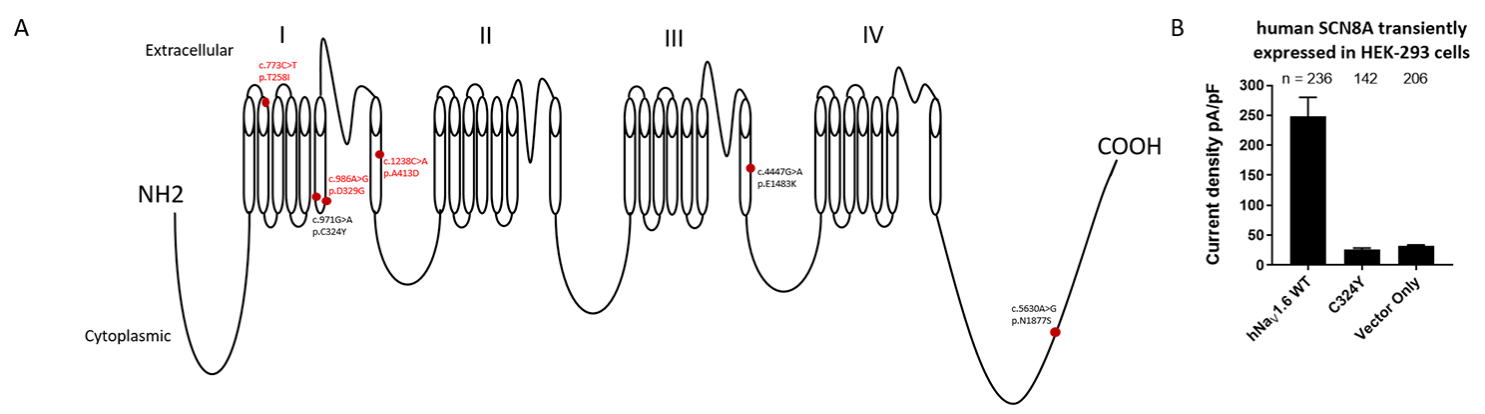
**A**. Simplified diagram of NaV1.6 channel showing the locations of the variants identified in our cohort (novel mutations are in red font). **B**. HEK-293 cells were transiently transfected with hNaV1.6 WT, hNaV1.6 C324Y, plasmid vector with no channel construct to look for functional effects of C324Y variant. C324Y peak current density (pA/pF) levels were significantly different from WT but not from Vector control

We studied eight patients from six families (males = 3, females = 5) with *SCN8A* heterozygous mutations. The phenotype ranged from DEE (n = 2), treatment responsive (n = 5) and an unclassified epilepsy phenotype, with possible clinical seizures in Patient 4. The age of seizure onset ranged from 3 months to 10 years. Individuals with DEE and an unclassified epilepsy phenotype presented with profound to severe intellectual disability and severe global developmental delay. Individuals with treatment responsive epilepsy were intellectually and developmentally within normal limits. Patient 4 had GDD and autism as a primary clinical phenotype with an abnormal EEG and possible clinical seizures. Treatment with valproic acid had improved EEG characteristics in the past. Four of them are seizure-free on monotherapy of carbamazepine and one with topiramate and clobazam. Exome sequencing identified three known and three novel heterozygous missense variations in *SCN8A.* Patient 4 also had a heterozygous, *de novo*, missense VUS in *KCNQ3.* Functionally, we observed a LoF, two GoF and three unclassified *SCN8A* variants. Electrophysiological analyses of the *SCN8A* variant in transfected cells revealed a LoF effect in Patient 4 (Fig. 1.).

## Discussion

*SCN8A* variants typically result in a moderate-severe epileptic encephalopathy, and account for 1% of the childhood epileptic encephalopathies [1]. The median age of seizures onset is typically 5 months (range: postnatal day 1 to 18 months of age) with multiple seizure types. The majority of affected patients have mild to severe global developmental delay. Abnormal tone, and abnormal movements may also be present [10]. In our cohort of eight individuals from six families with *SCN8A*-related disorders, we observed an age of onset ranging from 3 months to 10 years with severe to no clinical seizures. Developmental outcomes varied from profound developmental delay with intellectual disability and behavioural abnormalities to normal development. Developmental delay and age of onset of seizures did not seem to have a correlation in our cohort [11]. The seizure semiology in *SCN8A*-related disorders is variable, including focal seizures, tonic-clonic seizures, epileptic spasms, clonic seizures, absence, and myoclonic seizures [10, 12]. Patients with *SCN8A* mutations also have a high incidence of Sudden Unexpected Death in Epilepsy (SUDEP) [13, 14]. We noted a seizure course ranging from self-resolving focal seizures to Lennox-Gastaut syndrome (LGS) manifesting impaired awareness seizures, atypical absence seizures, generalized tonic-clonic seizures, epileptic spasms, and non-convulsive status epilepticus. The most common seizure type has been focal seizures as observed in the earlier reported patients [15].

The three novel variants are missense substitutions located on highly conserved transmembrane domains 1 and 2 of NaV1.6 (Fig. 1.). *SCN8A* gene variants causing substitution of amino acid residues in the highly conserved regions are often deleterious [1]. Three variants (those of Patient 2 [16, 17], Patient 3 [18], and Patient 4 [19]) were described previously. The clinical features of patient 2, and 3 were similar to what was previously described. Patient 4’s variant although published did not have phenotype information available for comparison. Variants in Patient 5 and Patient 6 have been submitted to ClinVar [20] without any detailed phenotype descriptions. It is important to note that individual differences in clinical manifestations can occur even with the same genetic variation.

LoF variants include an early stop-gain, indel frameshift or splice-site disruption resulting in truncated protein and reduced or abolished NaV1.6 function [21]. Missense changes causing GoF is the most common pathogenic mechanism for neuronal hyperexcitability and seizures. LoF is associated with cognitive impairment, movement disorders, and autism with or without seizures [22]. The clinical manifestations of *SCN8A* encephalopathy are likely reliant on the degree of GoF or LoF [23, 24]. GoF phenotypes include mild to severe epileptic encephalopathy. There are a few reported cases of benign or treatment-responsive infantile seizures with mild gain of function too [25]. We identified two GoF and a LoF variant with experimental evidence and three variations with unknown functional consequences. The electrophysiological analyses performed on Patient 4, LoF *SCN8A* variant (p.Cys324Tyr), offer valuable insights into the pathogenesis of *SCN8A*-related disorders. By characterizing the functional consequences of this variant, we provide evidence supporting its role in altering neuronal excitability and ion channel function. This information could potentially inform the development of targeted therapeutic strategies aimed at modulating ion channel activity to alleviate symptoms and improve patient outcomes.

In terms of the *KCNQ3* variant in Patient 4, this variant was found to be a conserved amino acid and all *in-silico* analyses suggest the variant has a deleterious impact; however, the variant is novel and remains a variant of uncertain significance. Functional validation has not been performed. Pathogenic variations in *KCNQ3* have been associated with benign or self-limited familial neonatal and infantile seizures (OMIM 121201) [26, 27]. Individuals are typically normal and grow out of their seizures, usually without any neurological sequalae in adulthood. More recently *KCNQ3* mutations are identified in patients with neurodevelopmental disorders and abnormal EEG [28]. Furthermore, alterations in this gene have been reported to act as risk factors for complex diseases including other epilepsy types and autism spectrum disorder. Sands et al. delineated an electroclinical phenotype in 11 patients with 4 different heterozygous *KCNQ3* GoF variants. Most of them did not have clinical seizures [28]. Patient 4 had EEG abnormalities with only possible clinical seizures which could plausibly be due to complex underlying molecular mechanisms involving *KCNQ3* and *SCN8A*.

Many early onset neurological diseases are now known to have a molecular basis. A genetic diagnosis can have strong implications for prognosis and treatment of epilepsy [29]. Assessments of how often a genetic diagnosis has clinically actionable implications vary from 20 to 60% [30, 31]. These comparisons highlight the variability in clinical presentations, epilepsy diagnoses, and genetic diagnoses among the patients with *SCN8A* pathogenic variations.

Intellectual disability, epilepsy, behavioral abnormalities, and movement disorders belong to a complex set of conditions with both monogenic and multifactorial etiologies. Clinical overlap between heterogeneous phenotypes, pleiotropy, variable penetrance, and expressivity makes genetic testing a huge challenge in these families. We describe a cohort of *SCN8A*-related disorders in this research work. The results of this study contribute to expanding the clinical and genotypic spectrum of *SCN8A*-related disorders. By identifying three novel variants in *SCN8A*, we have enhanced our understanding of the genetic landscape associated with these disorders. The observed variability in clinical presentation further emphasizes the complex nature of *SCN8A*-related disorders and highlights the need for personalized approaches to diagnosis, treatment, and genetic counseling. The functional data for p.Cys324Tyr confirms causation in *SCN8A*-related disorders.

## Conclusions

In conclusion, our study adds to the clinical and genotypic spectrum of *SCN8A*-related disorders by identifying novel variants and characterizing the functional consequence of p.Cys324Tyr. These findings underscore the importance of genetic testing in the diagnosis and management of individuals with *SCN8A*-related disorders. The mechanistic insights gained from this study may guide the development of targeted therapeutic interventions to improve patient care and outcomes in this heterogeneous group of disorders.

### Electronic supplementary material

Below is the link to the electronic supplementary material.

Supplementary Material 1

## Data Availability

The datasets generated and/or analysed during the current study are available in the ClinVar repository, https://www.ncbi.nlm.nih.gov/clinvar/ (Accession IDs: SCV004031478-SCV004031483).

## Abbreviations

OMIM: Online Mendelian Inheritance in Man
GDD: Global developmental delay
LoF: Loss-of-Function
GoF: Gain-of-Function
VUS: Variant of Uncertain Significance
EEG: Electroencephalogram
HEK-293: Human Embryonic Kidney cells
SUDEP: Sudden Unexpected Death in Epilepsy
LGS: Lennox-Gastaut Syndrome
WT: Wild-type
