# Supplementary material for: Expanding the genotype-phenotype spectrum in SCN8A-related disorders
Source: BMC Neurol. 2024 Jan 17;24:31. doi: 10.1186/s12883-023-03478-y (PMC10792783; doi:10.1186/s12883-023-03478-y)
Supplement: Supplementary file 1 — Supplementary Material 1 [file 12883_2023_3478_MOESM1_ESM.docx]

**Additional file 1**

Detailed case summaries of the patients in the study

**Case Reports**

**Patient 1**

A 17-year old young woman born to a non-consanguineous couple of Caucasian origin. She has *SCN8A*-developmental and epileptic encephalopathy, a hyperkinetic movement disorder, cerebral palsy, sleep disorder, neuromuscular scoliosis, severe intellectual disability and cortical visual impairment. A paternal cousin had epilepsy starting at 19 years of age and is currently seizure free. Mother is adopted and does not have any information about her biological family. The birth history was uneventful.

*Seizure disorder and treatment*

Epileptic (infantile) spasms (ES) were diagnosed at 4 months of age and the EEG showed hypsarrhythmia. ES were unresponsive to vigabatrin but responded to ACTH with normalization of the EEG. Epileptic spasms recurred on withdrawal of ACTH after 10 weeks treatment with recurrence of hypsarrhythmia. She continued to have ES and later developed tonic, atonic and atypical absence seizures. ACTH was retried followed by topiramate, nitrazepam. leveitracetam, valproic acid, prednisone and clonazepam. The ketogenic diet was associated with a reduction in clusters of ES from 7-10 per day to 1-5 clusters. Video-EEG monitoring at the age of 2 years and 9 months showed lack of posterior dominant rhythm and sleep potentials, multifocal delta and spikes. Tonic seizures and epileptic spasms were recorded. Phenytoin, oxcarbazepine and CBD were ineffective. The most recent video-EEG monitoring performed at 14 years and 17 years of age showed background changes and multifocal spikes. No clinical or subclinical seizures were recorded. She had hyperkinetic movements with no associated epileptiform changes. She is currently not having any clinical seizures and is on clonazepam.

*Development*

She has profound global developmental delay and severe intellectual disability. She was fixing and following by 3 months of age and smiled by 2 months of age. After the onset of epileptic spasms, her development regressed. She has no head control. She does not sit up independently. She recognizes her parents and smiles. She is non-verbal, wheelchair bound and G-tube fed.

*Neuroimaging*

Brain MRI at 2 years showed white matter volume loss and delayed myelination.

*Other testing*

Chromosomal microarray returned normal findings. Newborn screening and neurometabolic testing were normal. Exome sequencing revealed a novel *de novo* heterozygous variant in *SCN8A*, NM_001330260.2:c.1238C>A (p.Ala413Asp).

**Patient 2**

A 7-year old girl developmentally normal with familial infantile seizures due to a paternally inherited *SCN8A* mutation. Her parents are non-consanguineous couple of Caucasian origin. Her father (**Patient 2 - father**) was diagnosed with epilepsy in infancy at approximately 6 months of age, and responded to carbamazepine. At 5 years, carbamazepine was withdrawn and there was seizure recurrence. He was restarted on carbamazepine which he has been seizure free since 19 years of age.

*Seizure disorder and treatment*

At 3 months of age, she had an apparent life-threatening event characterized by sudden arching of back, stiffness, choking sounds, frothing, and greyish discoloration of the body followed by tiredness. At 6 months she had further episodes of limpness, shallow breathing and unresponsiveness and in some events had stiffening of her limbs and jerking of the right side of the body. She had 2-3 episodes per week lasting 2-6 minutes. EEG examination at 6 months was normal and events were not recorded. She was treated with carbamazepine and is seizure-free since except for a breakthrough seizure at 18 months of age with a missed dose of medication. Follow-up EEG at 15 months was normal.

*Development*

She is developmentally normal and performs well academically, reading prior to starting kindergarten. She has no attention or mood issues.

*Neuroimaging*

Brain MRI at 8 months of age was normal.

*Other testing*

Chromosomal microarray returned normal findings. Neurometabolic was normal. A paternally inherited heterozygous variant in *SCN8A,* NM_001330260.2:c.5630A>G (p.Asn1877Ser) was observed on exome sequencing. An incidental homozygous *BTD* variant was also found.

**Patient 2 - sibling**

A 4-year old boy with benign familial infantile seizures due to *SCN8A.* Maternal pre-eclampsia without complications was observed in his birth history as well.

*Seizure disorder and treatment*

His first episode of seizures occurred at 7 months characterized by body stiffening, oral automatisms, and grey colour lasting 3.5 minutes. He was started on carbamazepine and has remained seizure free since except for a breakthrough seizure at 18 months of age after a missed dose of medication. He is seizure-free on carbamazepine.

EEG examinations at 2.5 month showed bicentral temporal sharp transients during quiet sleep (performed neurology screening due to risk of seizures) and repeat EEG at one year was normal.

*Development*

He is developmentally normal and attends preschool.

*Neuroimaging*

Brain MRI at 8 months was normal.

*Other testing*

Paternally inherited well-known heterozygous variant in *SCN8A,* NM_001330260.2:c.5630A>G (p.Asn1877Ser) was observed, just like his sister (**Patient 2**).

**Patient 3**

Patient 3 is a 6-year old female with familial infantile seizures due to *SCN8A.* She was born to a consanguineous couple of Caucasian origin. Her father had febrile seizures and was on treatment with valproic acid until 4 years of age. Her birth history is uneventful.

*Seizure disorder and treatment*

Seizure onset was at 14 months of age, characterized by tonic stiffening with superimposed tremor, eye opening and unresponsiveness. She had post-ictal fatigue and irritability. A couple of them happened along with febrile illness. She had focal seizures occurring at a frequency of 2-4 a month lasting 2-3 minutes each. Her EEG at 18 months was normal. She was on topiramate and clobazam in the past. She is currently seizure-free off anti-seizure medication for over a year.

*Development*

She is developmentally normal.

*Neuroimaging*

Brain MRI at 2 years of age was normal.

*Other tests*

Chromosomal microarray returned normal findings. Exome sequencing identified known, *de novo*, heterozygous variant in *SCN8A*, NM_001330260.2:c.4447G>A (p.Glu1483Lys).

**Patient 4**

Patient 4 is an 8.5-year old female with global developmental delay, intellectual disability, autism spectrum disorder an abnormal EEG and possible clinical seizures. Pregnancy and birth history is uneventful. There is no family history of seizures.

*Seizure disorder and treatment*

At 4.5 years, episodes of zoning out with unresponsiveness with decreased truncal tone was noted. No other types of events were noted and seizures were never observed during EEGs. EEG at 7 months and 19 months were normal and at 2 years and 4 months showed left temporal sharp waves with a dipole configuration during sleep. At 4.5 years her EEG showed independent bilateral posterior temporal sharp waves and sharp and slow wave complexes but no clinical events. A decision was made to try valproic acid which did reduce the staring spells. A follow-up EEG at 6.5 years showed slow background and multifocal spikes, maximal in the bilateral posterior quadrants and at 7 years and 8 months showed fragmentary spike wave in the bilateral anterior quadrants. Valproic acid was stopped due to perceived negative impact on her behavior

*Development*

She has severe global developmental delay with intellectual disability. Development delay was first noted at 4 months of age when she had poor eye contact but no regression. She took first steps at 3 years pf age. Autism spectrum disorder was diagnosed at the age of 15 months and she has impulsive behavior and hyperactivity.

*Other tests*

Exome sequencing identified known, *de novo*, heterozygous variant in *SCN8A*, NM_001330260.2:c.971G>A (p.Cys324Tyr) and a novel, *de novo,* heterozygous variant in *KCNQ3*, NM_004519, c.1120C>G (p.Pro374Ala). Neurometabolic testing, hearing were normal.

**Patient 5**

Patient 5 is an 8-year old boy of Indian origin with global developmental delay, autism spectrum disorder, ADHD treatment resistant epilepsy and a hyperkinetic movement disorder. His mother’s pregnancy was complicated by gestational hypothyroidism, diabetes mellitus and hypertension.

*Seizure disorder and treatment*

His seizures started at 5 months of age characterized by arrest of activity with eyelid fluttering lasting 3-5 seconds, 1-2 times per day consistent with atypical absence seizures. EEG at 1.5 years showed frequent generalized atypical spike and wave activity maximal in the bilateral anterior quadrants during drowsiness and sleep Seizures responded clinically to ethosuximide and acetazolamide. The most recent EEG performed at 7 years of age showed 7 Hz posterior dominant rhythm and bursts of 4 Hz spike wave with no clinical signs of seizures.

*Development*

He has global developmental delay, autism spectrum disorder, intellectual disability and ADHD. Parents noticed delay in development around 3 months of age and poor eye contact. At 5 years he spoke about 20 words. . He has appropriate facial expressions but unable to perceive others’ emotions. He is shy and uncomfortable in social gatherings, does not initiate play with others. He is inattentive at pre-school. He first sat at 8-10 months, crawled at 1 year, walked at 2 year and he has an ataxic gait.

*Neuroimaging*

Brain MRI at 4 years showed mild nonspecific T2 hyperintense changes within the peritrigonal white matter.

*Other tests*

Exome sequencing identified heterozygous, *de novo*, known but unreported (ClinVar Variation ID 559632) variant in *SCN8A*, NM_001330260.2:c.773C>T (p.Thr258Ile).

**Patient 6**

Patient 6 is a 20-year old female of Indian origin with a hyperkinetic movement disorder and focal seizures.

*Seizure disorder and treatment*

Focal unaware seizures started at 10 years of age characterized by lack of aura, head and eye deviation to the left, face pulling to the left and altered awareness lasting 10-15 seconds. Her EEG at 14-years was normal. She is now seizure-free on carbamazepine.

*Development*

She is developmentally normal.

*Neuroimaging*

Normal

*Other tests*

Small echogenic abnormality found on renal ultrasound (?urachal remnant). Exome sequencing identified a heterozygous *de novo*, known but unreported variant (ClinVar Variation ID 1518887) in *SCN8A*, NM_001330260.2:c.986A>G (p.Asp329Gly).
